# Experimental Test of Reinforced Timber of FRCM-PBO with Pull-Off Adhesion Method

**DOI:** 10.3390/ma15217702

**Published:** 2022-11-02

**Authors:** Piotr Sokołowski, Paulina Bąk-Patyna, Dominika Bysiec, Tomasz Maleska

**Affiliations:** 1 Faculty of Civil Engineering and Architecture, Kielce University of Technology, Al. Tysiąclecia Państwa Polskiego 7, 25-314 Kielce, Poland; 2 Faculty of Civil Engineering and Architecture, Opole University of Technology, Katowicka 48, 45-061 Opole, Poland

**Keywords:** pull-off test, composite, timber, adhesion, Fiber Reinforced Cementitious Matrix, p-Phenylene benzobis oxazole

## Abstract

The article describes the results of pull-off adhesion strength of the FRCM-PBO (Fiber Reinforced Cementitious Matrix-p-Phenylene benzobis oxazole) composite adhered to the epoxy resin layer which is the connector with the timber beam. In addition, this paper shows the results of the tests of resistance to pull-off the epoxy resin layer from the pine beam. The tests were carried out based on the Polish Standard PN-EN 1542. The Pearson linear correlation analysis was also carried out in order to determine the correlation between the obtained results and the destructive forces. The factors that occurred during the test that may affect its results, such as the method of applying the bursting force, surface preparation of the tested elements and the types of substrate destruction, were also characterized. The experimental data show that in all the tested samples, non-initial adhesive destruction between the adhesive layer and the disc was observed.

## 1. Introduction

Many existing structures require strengthening and retrofitting. This aspect is very important due to the deterioration of structures, changes in using increasing design requirements, or functionality of these structures. In the case of concrete structures, the traditional methods of strengthening are still the most popular. In this case, the traditional materials such as concrete or steel are well known and very common in practical application. For several years, methods using carbon fiber reinforced polymer composites (CFRP) have been very popular. These materials exhibit great mechanical characteristics and high durability [1,2,3,4,5,6,7,8]. These methods of strengthening are adequate to methods from this paper, where the pull-off adhesion strength of the FRCM-PBO (Fiber Reinforced Cementitious Matrix-p-Phenylene benzobis oxazole) composite to timber was analyzed. Timber is one of the oldest building materials. It is a truly ecological material, fully renewable and healthy, and environmentally friendly as it takes carbon dioxide from the atmosphere. Its use in construction has a positive effect on unfavorable climate changes. As it is commonly known, timber is an anisotropic material, i.e., physical and mechanical properties change depending on the considered direction, which (in engineering terms) means the dependence of tensile, bending and compression strength on the direction of the force in relation to the direction of the fibers [9,10]. The timber shows the best mechanical parameters along the grain. In combination with its low structural density, timber structures are relatively light, which allows the construction of structures with large spans and heights. Timber constructions, however, are not without their drawbacks. Like other building materials, they require repair or strengthening as they age. According to Masłowski [11], the reinforcement of timber structures may result, among others, from atmospheric, chemical and biological factors, temperature changes, destructive activities of the environment, design and construction errors. Additionally, after a certain period of use the requirements of the user of the object may have changed and the current load-bearing capacity of the object is insufficient. Therefore, it is necessary to take appropriate measures to obtain a higher load capacity.

Currently, the traditional and proven methods of strengthening timber structures are willingly replaced by modern systems based on synthetic polymers [12,13,14,15,16,17,18,19]. Composites, which is the topic of this paper, were created as a result of the search for materials that would have better strength, physical and chemical parameters with greater strength than traditional materials, stiffness and possibly low density. This group is one of the most spectacular achievements of material engineering. Composite material (Latin compositus = complex) is a material that is made up of at least two different components that combine on a macroscopic level [20,21,22,23].

In FRP systems (Fiber Reinforced Polymers), the composite is joined with timber by means of a matrix made of epoxy resin, which is the link between the reinforced element and the reinforcement and is most often in the form of fibers. Unfortunately, the mechanical properties of the FRP system depend mainly on the temperature, and particularly on the glass transition temperature of the epoxy resin, in the range of 40–80 °C, which is what determines the durability and effectiveness of the reinforcement, regardless of the fibers used. When the glass transition temperature is exceeded, the matrix loses its properties and the FRP system becomes an ineffective reinforcement. In response to this problem, the FRCM (Fiber Reinforced Cementitious Matrix) system was developed, in which the resin matrix was replaced with a mineral mortar [24,25]. One of the most commonly used reinforcement materials in this system is a mesh made of PBO (p-Phenylene benzobis oxazole) fibers. However, this system is also burdened with imperfections resulting from the sliding effect occurring between the mineral mortar and the fibers. This is due to the mortar not able to cover all fibers exactly like epoxy resin. Thus, premature detachment of the fibers from the matrix takes place, which in turn leads to incomplete use of the mechanical properties of PBO fibers. This can be remedied by using additional PBO mesh anchors, which can prevent premature fiber detachment and thus increase the capacity utilization of the PBO mesh.

The article presents the results of tests on the pull strength of timber beams reinforced with FRCM-PBO composite. The PBO mesh determines the load-bearing capacity of the tension zone of the timber structure. The cooperation of the mesh and the mineral mortar in absorbing the external load is also of great importance. The mechanism of cooperation between the composite and the timber beam is the essence of the reinforced structure. This cooperation was ensured by the adhesion of the mesh and the surrounding mortar. The method of beam reinforcement occurs in three stages. In stage I, the tearing strength of the FRCM-PBO composite from the epoxy resin layer was determined, which was additionally roughened with quartz sand to ensure a better bond. In stage II, the resistance to pull-off the epoxy resin layer from the timber substrate was determined. In stage III, the Pearson linear correlation coefficient was determined to determine the correlation of the obtained results.

## 2. Description of Pull-Off Test

**Figure 1:**
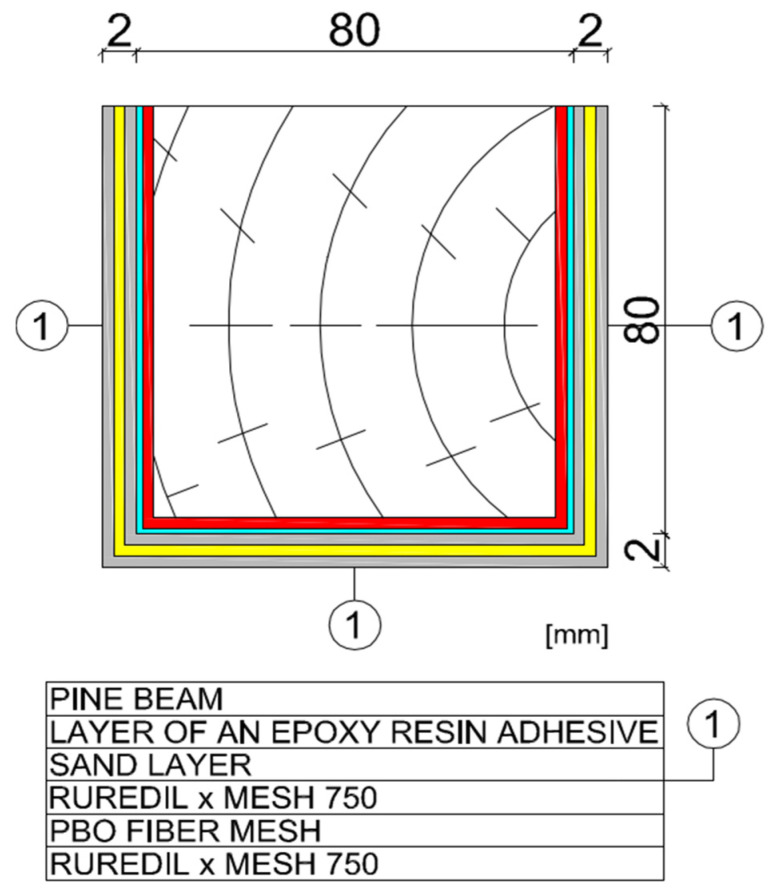
The strengthening a timber beam of research.

The adhesion pull-off test is a process used to determine how well a coating performs and bonds to a particular substrate, such as wood or concrete. This test is performed by gluing a loading point to the surface with an epoxy or polyester resin. After gluing, the loading force is measured between materials and loading point. In this paper the pull-off is a test carried out on the surface of a composite to determine the tensile strength of materials and adhesion of layers. The pull-off test was described, e.g., in the PN-EN 1542 standard “Products and systems for the protection and repair of concrete structures—Test methods—Measurement of adhesion by pull-off” [26]. According to the standard, the result of the test measurement was the value expressed in MPa, calculated from the force at which the layer of the previously cut material was torn off, to which the measuring plug breaking the sample was glued, in relation to the surface of the cut layer. The standard [14] provides 8 types of standard damage (Table 1) and indicates the cases when the measurement result should be rejected. The measurement is a fully correct result when the destruction (disc tearing off the tested material) occurs in the tested substrate Figure 1. 

## 3. Stage of Experimental Research

### 3.1. General Remarks

In this research, the depth of drilling was calculated according to the assumptions resulting from the formula specified in the standard [26]:

*d*_l_= *d_d_* + (15 ± 5), (1)

where:

*d_l_*—total drilling depth in [mm],

*d_d_*—layer thickness in [mm].

In the case of adhesion, strength of samples was calculated according to the formula

(2) $$f_{h}=\frac{4F_{h}}{\pi D^{2}},$$

where:

*f_h_*—adhesion strength of the sample in [Mpa],

*F_h_*—failure pull-off force in [Mpa],

*D*—average dimension of the sample in [Mpa].

**Figure 2:**
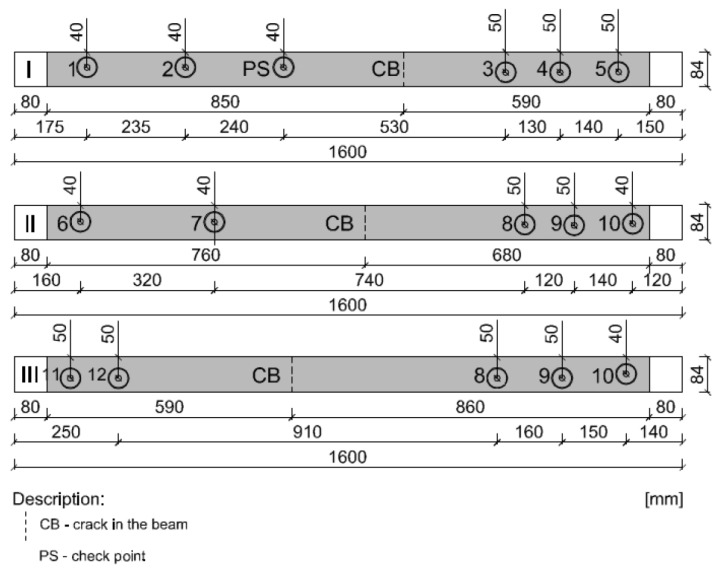
Distribution scheme of the tested samples.

In this research, the research samples were similar to each other in the three different stages. Detailed samples are shown in Figure 2. In addition, the surface of wood was prepared very precisely by careful sanding of samples, because in this research the measure of roughness was not possible.

### 3.2. Stage I of Pull-Off Test

#### 3.2.1. General Description of Stage I

**Figure 3:**
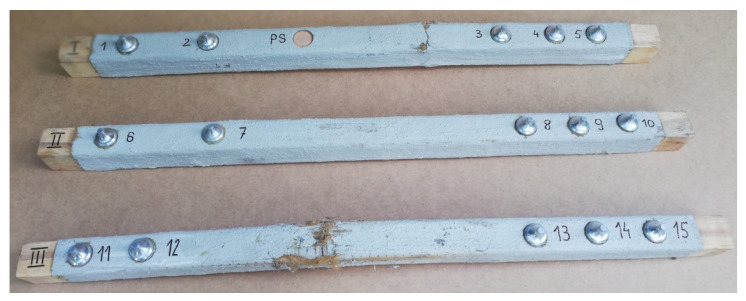
View of the tested samples for stage I.

For the first step, the pull-off strength of the epoxy resin layer, i.e., Resin 55 adhesive, from the timber substrate was determined. The first step was to clean the places of sticking the steel discs from the remnants of the mineral mortar to the layer of Resin 55 resin glue, (as shown in Figure 3) to a depth of 5 mm. The next step was to degrease the place of sticking the discs and to glue steel discs with a diameter of 50 mm directly into the surface of the tested material. The discs were glued with an adhesive based on Pattex epoxy resin. The thickness of the glue joint was 1 mm. The samples were placed in the same places as in stage I.

#### 3.2.2. Samples of Stage I

**Figure 4:**
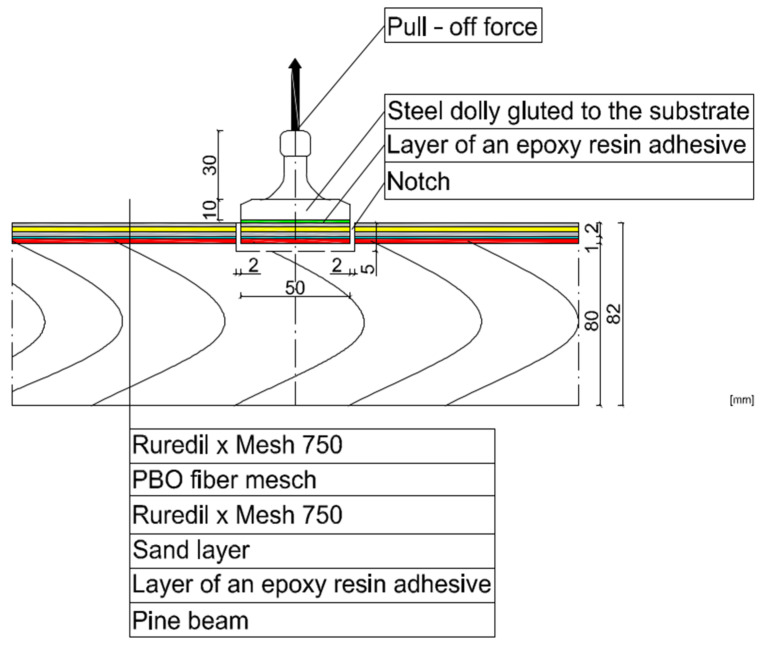
View of the tested samples from stage I.

Three timber beams made of pine with dimensions of 80 × 80 × 1600 mm, timber strength class C 24, reinforced with FRCM-PBO composite, glued to the layer of Resin 55 resin glue, which were additionally roughened with quartz sand, were used in the tests. The beam humidity ranged from 13.3 to 14.2%, while the density ranged from 0.59 to 0.61 g/cm^3^. Then, the composite layers were cut to a depth of 5 mm according to the assumptions of Formula (1) resulting from the standard [26]. After degreasing, steel discs with a diameter of 50 mm were glued with 5 Pattex glue on each of the beams (Figure 2 and Figure 3). The test stand included the PosiTest AT-M Adhesion Tester with the detachment force recording software. The measuring instrument was set at an angle of 90° to the drilled surface, preventing it from changing its position during the test. The load increased steadily and continuously at the rate of 0.05 MPa/s until failure occurred. The test scheme was presented in Figure 4.

### 3.3. Stage II of Pull-Off Test

#### 3.3.1. General Description of Stage II

In the second stage, the pull-off strength of the epoxy resin layer, i.e., Resin 55 adhesive, from the timber substrate was determined. The first step was to clean the places of sticking the steel discs from the remnants of the mineral mortar to the layer of Resin 55 resin glue (as shown in Figure 1 and Figure 2) to a depth of 5 mm. The glue Resin 55 (component A/component B) was characterized by the following properties: (i) viscosity at 22 °C of 10,400 cps/80 cps; (ii) tensile strength 36 MPa/72 MPa; (iii) tensile modulus 2220 MPa/2110 MPa; (iv) elongation at break 1.73%/5.89%; (v) flexural strength 63 MPa/126 MPa; (vi) flexural modulus 3660 MPa/3030 MPa; (vii) compressive strength 109 MPa/99 MPa; (viii) compressive modulus 2990 MPa/2690 MPa; (ix) Density 1.15 kg/L/1.15 kg/L. The next step was to degrease the place of sticking the discs and to glue steel discs with a diameter of 50 mm directly into the surface of the tested material. The discs were glued with an adhesive based on Pattex epoxy resin. The thickness of the glue joint was 1 mm. The samples were placed in the same places as in stage I.

#### 3.3.2. Samples of Stage II

**Figure 5:**
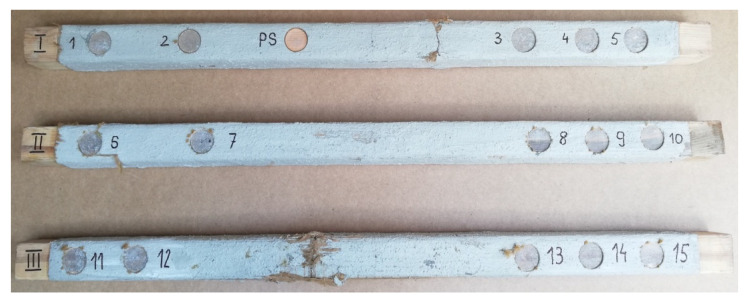
View of the tested samples from stage II.

**Figure 6:**
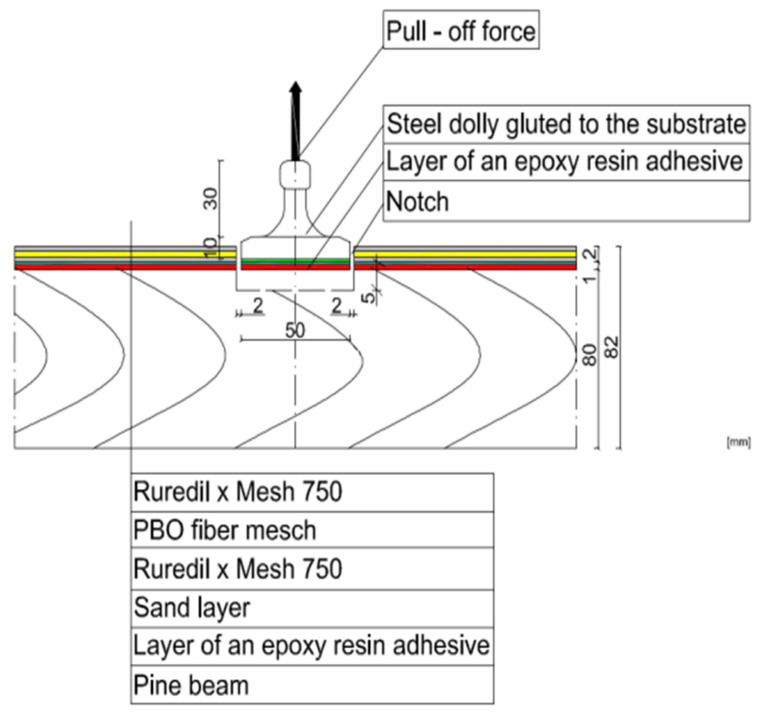
View of the tested samples for stage II.

After degreasing, 5 steel discs 50 mm in diameter were glued to each of the beams (Figure 5). The test stand included the PosiTest AT-M adhesion tester with the pull-off force recording software. The measuring instrument was set at an angle of 90° to the drilled surface, making it impossible to change its position during the test. The load increased continuously at a rate of 0.05 MPa/s until failure occurred. The test scheme was shown in Figure 6. The pull-off strength of the tested samples was calculated from the Formula (2).

## 4. Results from Experimental Research

### 4.1. Result of Stage I of Pull-Off Test

**Figure 7:**
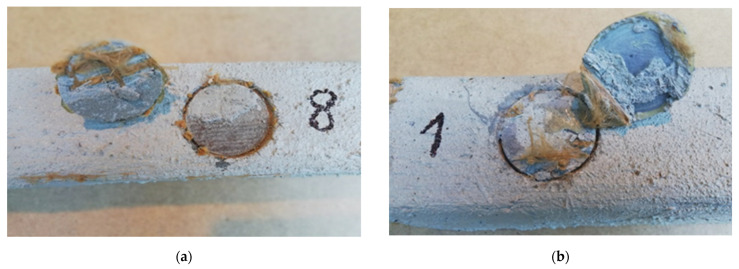
Y/Z—adhesive failure between: (**a**) the adhesive layer and the disc, (**b**) the adhesive layer and the disc 70%.

**Figure 8:**
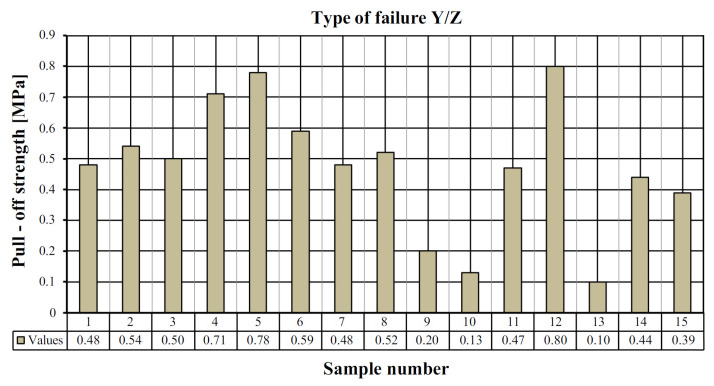
View of the tested samples for stage I.

Table 2 shows the pull-off strength values with a description of the destruction for individual tested samples. In all cases, adhesive failure was observed between the adhesive layer and the ‘Y/Z’ disc. In five cases, the disc was detached from the resin substrate, which resulted in no damage to the mineral layer of the composite, which the standard [26] defines as “Y/Z—adhesive damage between the adhesive layer and the disc” (Figure 7). In ten cases, there was a non-standard—partial destruction in the composite layer, which resulted in the disc detaching from the composite substrate. In this case, the percentage of the damaged area was determined visually. Based on the research, the percentage of the damaged area between the adhesive layer and the disc was estimated in the range from 5 to 70% (Figure 7). Table 3 presents a summary of the statistical analysis of the test results. Figure 8 shows the graphical representation of the pull-off strength for the obtained failure type ‘Y/Z’.

### 4.2. Results of Stage II of Pull-Off Test

**Figure 9:**
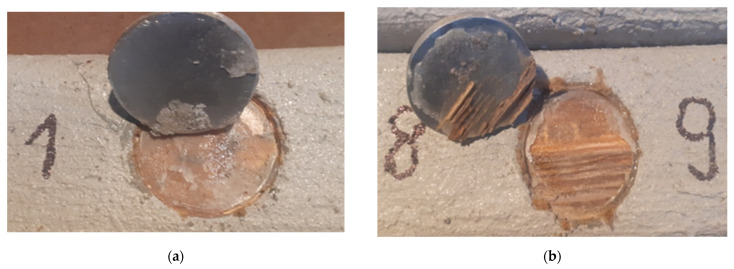
Y/Z—adhesive failure between: (**a**) the adhesive layer and the disc, (**b**) the adhesive layer and the disc 70%.

**Figure 10:**
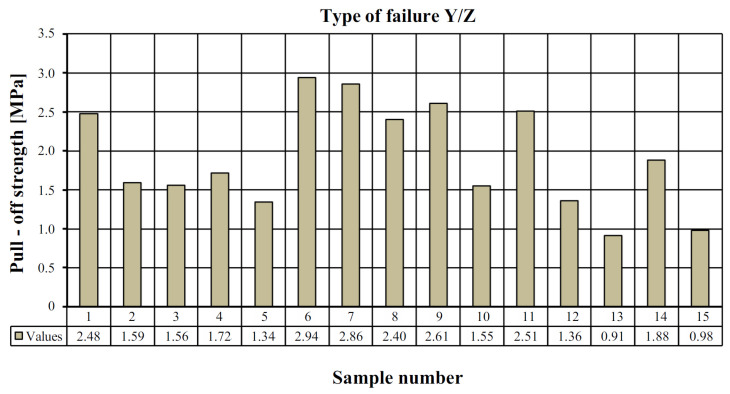
The values of the pull-off strength for the type of substrate failure “Y/Z”.

Table 4 shows the pull-off strength values with a description of the destruction for individual tested samples. As before, in all cases, adhesive failure was observed between the adhesive layer and the ‘Y/Z’ disc. In eight cases, the disc detached from the resin substrate, which resulted in no damage to the mineral layer of the composite, for which the standard is [26] defined as “Y/Z—adhesive damage between the adhesive layer and the disc” (Figure 9). In seven cases, there was a non-standard—partial destruction in the composite layer, which resulted in the disc detaching from the composite substrate. In this case, the percentage of the damaged area was determined visually. Based on the research, the percentage of the damaged area between the adhesive layer and the disc was estimated between 5 and 90% (Figure 9). Table 5 presents a summary of the statistical analysis of the test results. Figure 10 shows the graphical representation of the pull-off strength for the obtained failure type ‘Y/Z’.

### 4.3. Determination of Pearson’s Linear Correlation Coefficient

As mentioned earlier, the beams in question were subjected to a destructive test [27], and the test results of which, together with the arithmetic mean of the pull-off strength for stages I and II, are shown in Table 6.

The determination of the Pearson linear correlation coefficient was to determine the correlation between the destructive force *F_max_* and the pull strength value for stages I and II. For this purpose, the formula was used [28]:

(3) $$r_{xy}=\frac{\sum^{\;}\left(X_{i}\right)-\left(\bar{X}\right)\times \left(Y_{i}\right)-\left(\bar{Y}\right)}{\sqrt{\sum^{\;}\left(X_{i}\right)-({\bar{X)}}^{2}\times }\sum^{\;}\left(Y_{i}\right)-({\bar{Y)}}^{2}\;}=\frac{\frac{1}{n}\sum^{\;}X_{i}Y_{i}-\bar{X}-\bar{Y}}{\;\sigma X\;\sigma Y\;}=\frac{cov\;XY}{\sigma X\;\sigma Y}$$

where:

*X_i_*, *Y_i_*—observation values (X—stage I, Y—stage II),

$\bar{X}$, $\bar{Y}$—population averages X and Y,

*σX*, *σY*—population averages X and Y,

*cov*(x,y)—covariance of variables X and Y,

*n*—number of observations.

For the relationship, the breaking strength of the pull-off strength for stage I, the Pearson linear correlation coefficient “*r*” was positive and amounted to 0.535. This means a strong relationship between the examined features, i.e., with an increase or decrease in destructive forces, the tear strength increases or decreases analogously.

**Figure 11:**
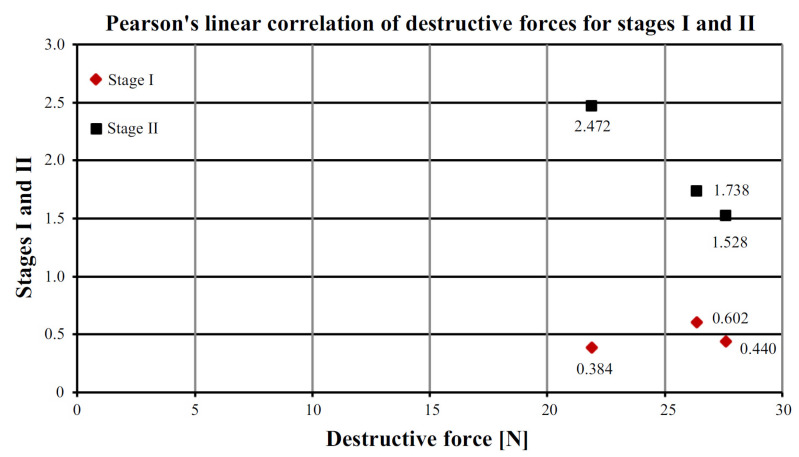
Pearson’s linear correlation for destructive forces and pull-off strength for stages I and II.

For the relationship, the force breaking the peel strength for stage II, the Pearson linear correlation coefficient “*r*” was negative and amounted to −0.999. This means that as the destructive forces increase or decrease, the pull-off strength behaves in the opposite way and decreases or increases accordingly. The dependence of the force destroying the breaking strength of the Pearson linear correlation for stages I and II was shown in Figure 11.

**Figure 12:**
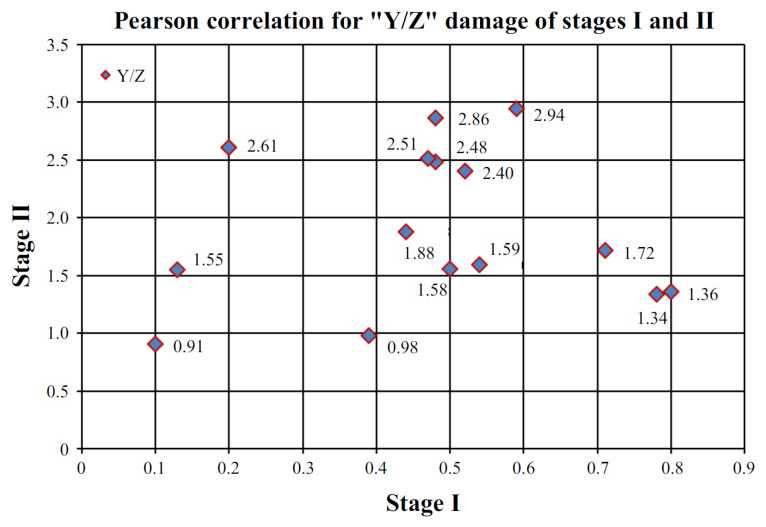
Pearson’s linear correlation for the “Y/Z” failure types for stages I and II.

The correlation coefficient “*r*” was calculated in the same way for stages I and II, which was 0.0534. This means that there was no correlation between Stage I and Stage II results. The plot of Pearson’s linear correlation for the “Y/Z” failure types was shown in Figure 12.

## 5. Conclusions

The article discusses the results of pull-off adhesion strength of the FRCM-PBO composite adhered to the epoxy resin layer which was the connector with the wooden beam, and the results of the tests of resistance to peeling off the epoxy resin layer from the pine beam. The linear Pearson correlation analysis was also carried out in order to determine the correlation between the obtained results and the destructive forces. The tests were carried out based on the Polish Standard PN-EN 1542 “Products and systems for the protection and repair of concrete structures—Test methods—Measurement of adhesion by tearing off”. The obtained results show that in both stages, in all tested samples, there was an adhesive failure between the adhesive layer and the disc, defined by the standard [26] as “Y/Z”. In addition, the following conclusions can be drawn:

- The adhesion of the adhesive used to the metal disc was too low to detach the entire FRCM-PBO composite from the resin and wood substrates;
- Construction of the FRCM composite consisting of a mineral layer and PBO fibers had a positive impact on the results, because the wooden beams were strengthened. Finally, their load capacity was increased;
- During the tests, the effect of crumbling of the composite mineral matrix and separation of PBO fibers was observed. The result was only one type of destruction—“Y/Z”. Another reason may be that the incision is too small (five millimeters) although it is in accordance with the standard [26]. In a previous paper [29], cuts 10 mm deep were used, and the standard [30] recommendation is to make an incision even 15 mm deep on the sample surface;
- Pearson’s linear correlation coefficient for stages I and II showed no correlation between the obtained results. This means there is no linear relationship between the studied variables. This situation was a kind of surprise, because the dependence between the examined features was expected, and more-so that a positive and negative correlation was found between the destructive force and the pull-off strength values of stages I and II;
- According to the standard [26], the values of the failure type “Y/Z” should be rejected. The obtained results indicate a good bond between the FRCM-PBO composite and the epoxy resin layer as well as between the epoxy resin layer and the tear-off wooden beam. This is evident by the fact that in order to de-tach the metal disc from the substrates used, a greater detachment force is needed than the values obtained during the tests.

Generally, for stage I, the depth greater than 5 mm would not significantly affect the obtained results but would significantly affect the obtained results in the case of stage II. Therefore, in future studies of this type, a deeper incision should be considered. The key facts that also influence the adhesion is the roughness of the surface and the thickness of the joint of the adhesive used, which in this case was 1 mm. Thus, in the future the authors plan to develop this field of research.

## Figures and Tables

**Table 1:** Types of failures.

Type of Failure	Description of Failure
A	concrete substrate—cohesion failure
A/B	substrate and the first layer—adhesion failure
B	first layer—cohesion failure
B/C	first and second layer—adhesion failure
C	second layer—cohesion failure
−/Y	last layer and adhesive layer—adhesion failure
Y	adhesive layer—cohesion failure
Y/Z	adhesive layer and the dolly—adhesion failure

**Table 2:** Pull-off strength with description of sample failure for stage I.

Beam Number	Sample Number	Pull-Off Strength [MPa]	Sample Location Description
I	1	0.48	Y/Z—adhesive failure between the adhesive layer and the disc 40%
	2	0.54	Y/Z—adhesive failure between the adhesive layer and the disc
	3	0.50	Y/Z—adhesive failure between the adhesive layer and the disc
	4	0.71	Y/Z—adhesive failure between the adhesive layer and the disc 10%
	5	0.78	Y/Z—adhesive failure between the adhesive layer and the disc 15%
II	6	0.59	Y/Z—adhesive failure between the adhesive layer and the disc 40%
	7	0.48	Y/Z—adhesive failure between the adhesive layer and the disc
	8	0.52	Y/Z—adhesive failure between the adhesive layer and the disc 30%
	9	0.20	Y/Z—adhesive failure between the adhesive layer and the disc 5%
	10	0.13	Y/Z—adhesive failure between the adhesive layer and the disc 10%
III	11	0.47	Y/Z—adhesive failure between the adhesive layer and the disc
	12	0.80	Y/Z—adhesive failure between the adhesive layer and the disc 70%
	13	0.10	Y/Z—adhesive failure between the adhesive layer and the disc
	14	0.44	Y/Z—adhesive failure between the adhesive layer and the disc 30%
	15	0.39	Y/Z—adhesive failure between the adhesive layer and the disc 20%

**Table 3:** List of the statistical data of the test results (stage I).

Type of Failure	“Y/Z”
Average pull-off strength [MPa]	0.47
Standard deviation [MPa]	0.21
Coefficient of variation	0.45
Standard error	0.054
Minimum	0.1
Maximum	0.8
Sum	7.13
Range	15

**Table 4:** Pull-off strength with description of sample failure for stage II.

Beam Number	Sample Number	Pull-Off Strength [MPa]	Sample Location Description
I	1	2.48	Y/Z—adhesive failure between the adhesive layer and the disc
	2	1.59	Y/Z—adhesive failure between the adhesive layer and the disc
	3	1.56	Y/Z—adhesive failure between the adhesive layer and the disc
	4	1.72	Y/Z—adhesive failure between the adhesive layer and the disc
	5	1.34	Y/Z—adhesive failure between the adhesive layer and the disc
II	6	2.94	Y/Z—adhesive failure between the adhesive layer and the disc 80%
	7	2.86	Y/Z—adhesive failure between the adhesive layer and the disc 20%
	8	2.40	Y/Z—adhesive failure between the adhesive layer and the disc
	9	2.61	Y/Z—adhesive failure between the adhesive layer and the disc 10%
	10	1.55	Y/Z—adhesive failure between the adhesive layer and the disc
III	11	2.51	Y/Z—adhesive failure between the adhesive layer and the disc 90%
	12	1.36	Y/Z—adhesive failure between the adhesive layer and the disc 45%
	13	0.91	Y/Z—adhesive failure between the adhesive layer and the disc 5%
	14	1.88	Y/Z—adhesive failure between the adhesive layer and the disc 5%
	15	0.98	Y/Z—adhesive failure between the adhesive layer and the disc

**Table 5:** List of the statistical data of the test results (stage II).

Type of Failure	“Y/Z”
Average pull-off strength [MPa]	1.91
Standard deviation [MPa]	0.67
Coefficient of variation	0.35
Standard error	0.17
Minimum	0.91
Maximum	2,94
Sum	28.69
Range	15

**Table 6:** List of test results for beams I, II, III with arithmetic means of the tensile strength of stages I and II.

Beam Number	Destructive Force *F_max_* [N]	Arithmetic Mean Stage I	Arithmetic Mean Stage II
I	26.35	0.602 (samples 1–5)	1.738 (samples 1–5)
II	21.88	0.384 (samples 5–10)	2.472 (samples 5–10)
II	27.59	0.440 (samples 10–15)	1.528 (samples 10–15)

## Data Availability

Not applicable.
